# Feeling Oneself Requires Embodiment: Insights From the Relationship Between Own-Body Transformations, Schizotypal Personality Traits, and Spontaneous Bodily Sensations

**DOI:** 10.3389/fpsyg.2020.578237

**Published:** 2020-12-23

**Authors:** George A. Michael, Deborah Guyot, Emilie Tarroux, Mylène Comte, Sara Salgues

**Affiliations:** ^1^University of Lyon, Lyon, France; ^2^University Lyon 2, Laboratoire EMC (Cognitive Mechanisms Research Laboratory) (EA 3082), Lyon, France; ^3^University Lyon 2, Institute of Psychology, Lyon, France

**Keywords:** spontaneous sensations, embodiment, mental own-body transformations, schizotypy, schizotypal personality

## Abstract

Subtle bodily sensations such as itching or fluttering that occur in the absence of any external trigger (i.e., spontaneous sensations, or SPS) may serve to locate the spatial boundaries of the body. They may constitute the normal counterpart of extreme conditions in which body-related hallucinations and perceptual aberrations are experienced. Previous investigations have suggested that situations in which the body is spontaneously experienced as being deformed are related to the ability to perform own-body transformations, i.e., mental rotations of the body requiring disembodiment. We therefore decided to consider whether the perception of SPS might relate to embodiment as assessed through (i) the ability to perform own-body transformations (OBT task) and (ii) schizotypal traits (Schizotypal Personality Questionnaire, or SPQ), since high degrees of schizotypy in the general population have been associated with more vivid perceptions and aberrant perceptual experiences. Then participants completed a standard SPS task. Our analysis revealed that the slower the response time in the OBT task, the more frequent the perception of SPS. This suggests that difficulties in disembodying and mentally transforming one’s own body facilitate *feeling* oneself. Furthermore, a greater number of correct responses in the OBT task was associated with less frequent perception of SPS. This suggests that finding it easier to disembody and perform mental own-body transformations interferes with the ability to sense oneself. The results also show that higher schizotypal traits, as assessed through the SPQ, are associated with more frequent perception of SPS. Taken together, these results provide a coherent picture and suggest that embodiment is required in order to correctly *feel* oneself, as expressed through the perception of SPS. The ability to easily experience disembodiment reduces the sense of feeling oneself, and proneness to schizotypal traits produces body misperceptions that enhance and amplify this feeling. The results are discussed in the light of current knowledge and theories about body representations, taking into account attention and interoception as factors that influence body awareness. We offer explanations for perceptual aberrations, body-related delusions, and hallucinations based on misperceived or misinterpreted SPS, and we discuss possible mechanisms that may contribute to *feeling* and misperceiving oneself.

## Introduction

How does it feel to be you? How do you know that this is you? Subjective feelings of the sense of self, when one knows that this is one’s own hand or face (Gallagher, 2000), are grounded within one’s own body and representations of the body and its parts (Kinsbourne, 1998; Gallace and Spence, 2010; Sakson-Obada et al., 2018). This is what is known as embodiment, or embodied sense of self (Asai et al., 2016).

Research on embodied sense of self has garnered considerable interest during recent decades, and several experimental paradigms have been used to investigate its functional and neural aspects (Manos Tsakiris, 2010). Embodiment has primarily been investigated through its motor and behavioral aspects, such as sense of agency, i.e., subjective awareness of initiating, executing, and controlling voluntary actions (Braun et al., 2018); its sensory aspects, such as experimentally induced illusions that an external object is part of one’s body (e.g., the rubber hand illusion) (M. Tsakiris et al., 2007) or the measurement of the boundaries of the peripersonal space (e.g., the estimation of the moment a stimulus will be in collision with the body) (Canzoneri et al., 2012); own-body mental transformations, such as mentally rotating one’s own body in order to match a visually presented figure (Bonda et al., 1995; Blanke, 2005) and so on. All these paradigms (as well a number of others) are based primarily on responses to external triggers aimed at assessing self-related phenomena. Yet the term “sense of self” also evokes the status of one’s own body, which makes bodily sensations seem unique to oneself. This is the *feeling* that this body or a part of the body belongs to me (Gallagher, 2000; Braun et al., 2018;; Manos Tsakiris, 2017). Major contributions to our understanding of this aspect of embodiment come from research that involves the perception of natural bodily signals, i.e., interoception [(Bud) Craig, 2003; (Bud) Craig, 2004; Duschek et al., 2015]. This aspect is usually investigated through the perception of visceral signals such as heartbeats (Schandry, 1981) or gastric functions (Herbert et al., 2012), which is reminiscent of the original terminology (i.e., “enteroception,” meaning the perception of visceral sensations) (Vaitl, 1996). Since the above-mentioned paradigms are all meant to investigate embodiment and the sense of self, they should in theory yield consistent results overall. However, the picture is rather puzzling. For example, some researchers have found no clear relationship between heartbeat perception and the rubber hand illusion, while others have, and still others have found a relationship under certain circumstances only (Suzuki et al., 2013; Filippetti and Tsakiris, 2017; Crucianelli et al., 2018).

Imagine we try to conjure up a quick, introspective view of ourselves, sitting on a chair. Do we have to act, bump into objects, perform mental acrobatic rotations of our own body image, or feel visceral signals coming from inside to sense that we are there, having the direct and immediate experience of our own body in the first-person perspective? Sense of self does not seem to arise exclusively from the perception of stimuli delivered physically on peripheral receptors, the sense of agency, and the perception of visceral signals. Maybe a starting point to better apprehend some of the factors that contribute to the sense of self is looking at what underlies the perception of body boundaries and location in space, something that is clinical in nature (Stanghellini et al., 2014; Sakson-Obada et al., 2018).

The experience of abnormalities of consciousness and neuropsychiatric symptoms is considered by some researchers to be due to disruptions of the sense of self (Moe and Docherty, 2014) and the blur or malleability of the self-others boundaries (Thakkar et al., 2011; Noel et al., 2017; Di Cosmo et al., 2018). There is clinical evidence that abnormal functioning of some cortical areas, such as the temporoparietal junction, may result in seeing oneself from a distance (Blanke and Mohr, 2005), feeling a presence nearby (Arzy et al., 2006), or other bodily illusions (Leker et al., 1996; Persinger, 2001; Thakkar et al., 2011). In schizophrenia spectrum disorders, numerous symptoms relating to the body are observed (Chapman et al., 1978; Peled et al., 2000; Stanghellini et al., 2012), ranging from abnormal feelings of altered shape, to changes in the composition of the body and body decomposition, to blurred body boundaries (Noel et al., 2017; Di Cosmo et al., 2018; Sakson-Obada et al., 2018; Ferroni et al., 2019) up to the point that some authors suggested that schizophrenia (and related spectrum disorders) is a self-disorder in the sense that the first person perspective of the world is altered (Sass and Parnas, 2003). These disruptions in the basic sense of embodiment are viewed as key symptoms and are even used as early predictors of disease (Stanghellini et al., 2012; Ferri et al., 2014). These kinds of perceptual aberrations are also reported by individuals in the general population with high schizotypal personality traits (Chapman et al., 1978; Mohr et al., 2006; Arzy et al., 2007; Di Cosmo et al., 2018). Schizotypal personality refers to a set of traits thought to reflect the subclinical expression of the symptoms of schizophrenia spectrum disorders (Barrantes-Vidal et al., 2015) according to the theory of a psychosis continuum (Nelson et al., 2012). Individuals with high schizotypal traits are at higher risk for psychosis. Three types of factors seem to underlie behavior in schizotypy: cognitive-perceptual (ideas of reference, magical thinking, unusual perceptual experiences, and paranoid ideation), social-interpersonal (social anxiety, no close friends, constricted affect and, again, paranoid ideation), and disorganized (odd behavior and odd speech) (Raine et al., 1994). High schizotypal personality traits—especially in terms of cognitive-perceptual and social-interpersonal factors (Mohr et al., 2006; Van Doorn et al., 2018)—are associated with a higher frequency of body-related aberrant perceptual phenomena (Cicero et al., 2020), such as feelings of a presence nearby, spontaneous disembodiment and out-of-body experiences, perceptions that others fail to experience, modified perception of body parts, exaggerated self-consciousness (Sass and Parnas, 2003) and blurred body boundaries (Sass and Parnas, 2003; Ettinger et al., 2015; Di Cosmo et al., 2018; Ferroni et al., 2020). People with high schizotypal personality traits also seem to experience difficulties in perspective-taking (Langdon and Coltheart, 2001), which is also a basic feature of self-referential processes (Buckner and Carroll, 2007). These experiences suggest that schizotypal personality is associated with proneness to experience self-related disturbances (Mohr et al., 2006; Arzy et al., 2007) and, therefore, that schizotypy may be a way to investigate the sense of self and embodiment.

On the other hand, reports of body-related hallucinatory phenomena in neurologic and psychiatric populations, such as sensing a ghostly gentle touch or the feeling that insects are crawling beneath the skin (Berrios, 1982), suggest that disturbances of body awareness and the self may occur due to abnormal spontaneous activity in cortical areas related to somatosensation and disorders of multimodal integration (Blanke, 2005; Blanke and Arzy, 2005; Geoffroy et al., 2014; Eccles et al., 2015; Graziano, 2018; Bretas et al., 2020). Have you ever felt tingling, tickling, or other sensations on your skin even when there are no mosquitos around to bother you? There are situations in which awareness of touch occurs without any stimulus actually being delivered. Does this mean that you are experiencing some kind of body-related disturbance? These kinds of bodily sensations have been anecdotally reported in the scientific literature by clinical researchers working on tactile sensitivity, and are referred to as paresthesia (Schmidt et al., 1990a,b) or tactile hallucinations (Gallace and Spence, 2014). However, both these terms are highly evocative of pathological conditions (Berrios, 1982) and do not account for the fact that almost everyone experiences such sensations on an everyday basis. This is why the term “spontaneous sensations” (SPS) is preferred (Michael and Naveteur, 2011), as these are believed to constitute the normal counterpart of bodily hallucinations. The spontaneous nature of these phenomena does not allow for experimental manipulation at will. However, over the past 15 years, several researchers have used subjective reports to assess SPS under controlled conditions [e.g., (Naveteur et al., 2005; Michael and Naveteur, 2011; Bauer et al., 2014a; Tihanyi and Köteles, 2017)].

That SPS may constitute the ever-present background of bodily sensations through which body representations arise (Kinsbourne, 1998) suggests that their perception gives cues as to the position of body parts at a given moment, their spatial boundaries and limits, and that they therefore contribute to the sense of self (Michael et al., 2017; Sakson-Obada et al., 2018). The relationship between SPS and neurocognitive body-related processes has been established by means of several forms of evidence. For instance, neuroimaging findings suggest that attending to well-circumscribed and localized SPS results, as a minimum, in activation in the primary somatosensory cortex (Bauer et al., 2014a,b), which includes a precise point-by-point representation of the body (i.e., somatotopy) (Penfield and Boldrey, 1937). A recent investigation gathered evidence that default oscillatory EEG activity in both the primary and secondary somatosensory cortices relates to the perception of SPS, and probably reflects individual differences in bodily awareness. Furthermore, behavioral evidence also supports the involvement of somatosensory cortices in the perception of such sensations, since when perceived in the hands they exhibit a proximodistal gradient: SPS occur more frequently toward the fingertips. This spatial pattern strikingly resembles traces of peripheral receptor distribution in the somatosensory cortex (Schady et al., 1983). Another study found a relationship between SPS and EEG connectivity between cortical areas involved in self-awareness, such as the temporo-parietal junction and the insula (Salgues et al., 2021). Despite such cues, and despite suggestions that SPS may contribute to body awareness and the perception of the spatial limits and boundaries of the body, direct evidence of the relationship between SPS and embodiment is limited. In fact, SPS have been found to be related to interoception (Michael et al., 2015) as classically assessed through the silent heartbeat counting task (Schandry, 1981). Good heartbeat perceivers have increased perception of SPS, and this supports the idea that SPS have something to do with the sense of self and embodiment. Finally, chronic pain syndromes that modify the perception of the body through disruptions of somatosensory and interoceptive functions (Kim et al., 2017; Borg et al., 2018) also modify the perception of SPS (Borg et al., 2015; Echalier et al., 2020).

However, the picture is still incomplete. By nature and by definition, perceiving SPS is *feeling* oneself. Yet evidence of the relationship between SPS and other phenomena related to embodiment (or disembodiment) is missing. One way to establish such a relationship would be to show that SPS perception relates to performance in one of the tasks designed to assess self-referential processes, as would showing that proneness to experiencing body-related unusual phenomena changes the perception of SPS. Therefore, we set out to investigate the relationship between SPS and embodiment through a two-fold procedure. First, we used an own-body mental rotation paradigm (i.e., an own-body transformation task, or OBT task) (Blanke, 2005), in order to establish a direct relationship between SPS and the ability to change perspective by mentally rotating own-body image. The ease with which this mental transformation is achieved naturally relates to loose embodiment. If SPS were linked to embodiment, then increased performance in the OBT task would negatively correlate with SPS perception. Second, we used a popular self-completed schizotypal personality questionnaire (SPQ; Raine, 1991). As described above, individuals from the general population with high schizotypal traits have difficulties controlling perspective-taking (Langdon and Coltheart, 2001) and are more prone to experience sensations that others do not usually experience, or experience usual experiences more vividly (Chapman et al., 1978; Raine et al., 1994; Van Doorn et al., 2018). Therefore, if SPS were linked to embodiment, then schizotypal (especially cognitive-perceptual and social-interpersonal) traits would correlate with SPS perception, signaling either diminished self-perception or, on the contrary, exaggerated, enhanced and more vivid self-perception. In order to achieve our aim, all participants completed the SPQ, a validated SPS task, and a computerized OBT task.

## Materials and Methods

### General Procedure

The study was completed in two stages, separated by one to two weeks. During the first stage, participants completed the Edinburgh Handedness Inventory (Oldfield, 1971), the Schizotypal Personality Questionnaire (Raine, 1991), and a form containing questions on age, gender, height, weight, use of psychotropic medications (antidepressants, anxiolytics, neuroleptics, anticonvulsants, hypnotics, tranquilizers, etc.) and reason for use (if applicable), regular use of other psychoactive substances (alcohol, marijuana, etc.), and history of cardiovascular disease or diabetes. During the second stage, participants completed the SPS task, followed by the OBT task.

### Participants

The study was carried out in accordance with the Declaration of Helsinki and was approved by the local ethics committee (reference number IRB 00009118). Participants were undergraduate students at Lyon 2 University, France. Exclusion criteria were as follows: history of neurological or psychiatric disease, history of psychoactive substance abuse, history of diabetes or cardiovascular disease, a laterality quotient of less than 50%, reported body mass index (BMI) of less than 18 and greater than 31 kg/m^2^ (previous research showed that BMI influences interoceptive processes and SPS, this is why it is necessary to control for such a confounding factor; (Cameron, 2002; Michael and Naveteur, 2011; Michael et al., 2015)], incomplete responses to the SPQ, or more than 50% errors in the OBT task. Of the 78 individuals that initially volunteered to participate, 23 were excluded on the basis of the above-mentioned criteria or did not show up to complete the second stage of the research. The sample therefore included 55 participants (44 female and 11 male), all right-handers (mean laterality quotient 88.7, SD = 13.7), with a mean age of 21.3 years (SD = 2.03; range: 18 to 27) and a mean BMI of 21.4 kg/m^2^ (SD = 1.94; range: 18.1 to 27 kg/m^2^). Power analyses were conducted on the effect size of the proximo-distal gradient in frequency of SPS, which is their standard characteristic. Based on 8 published studies (Michael and Naveteur, 2011; Michael et al., 2012, 2015, 2017; Beaudoin and Michael, 2014; Borg et al., 2015; Echalier et al., 2020; Salgues et al., 2021) carried out on 367 participants (10 experiments and 1206 hand maps), the weighted mean affect size expressed as Cohen’s *w* is 0.38 (i.e., medium to large) and expressed as Cramér’s *V* is 0.20 (i.e., medium to large). Provided a power of 90% to detect a medium effect size, the number of hand maps needed is 99. Given that two hand maps per participants are collected (one per hand) in the present study, the sample size needed is 50 participants. With a sample of 55 participants (i.e., 110 hand maps) the power is good (92%) and the probability that Type II errors occur is considerably reduced. All participants gave their written informed consent to participate prior to the test.

### SPQ

The Schizotypal Personality Questionnaire (Raine, 1991) is a commonly used self-report measure of schizotypy in healthy community samples. It is a simple and quick evaluation involving 74 quoted items with a “yes/no” response, exploring the DSM-IV criteria for schizotypal personality disorder. Its structure reveals a three-factor model of schizotypy reflecting positive/cognitive-perceptual symptoms, negative/social-interpersonal symptoms, and disorganization. The French version of the SPQ (Dumas et al., 2000) has the same factorial structure as the original, a high internal reliability, and good internal consistency.

#### SPS Task

**FIGURE 1:**
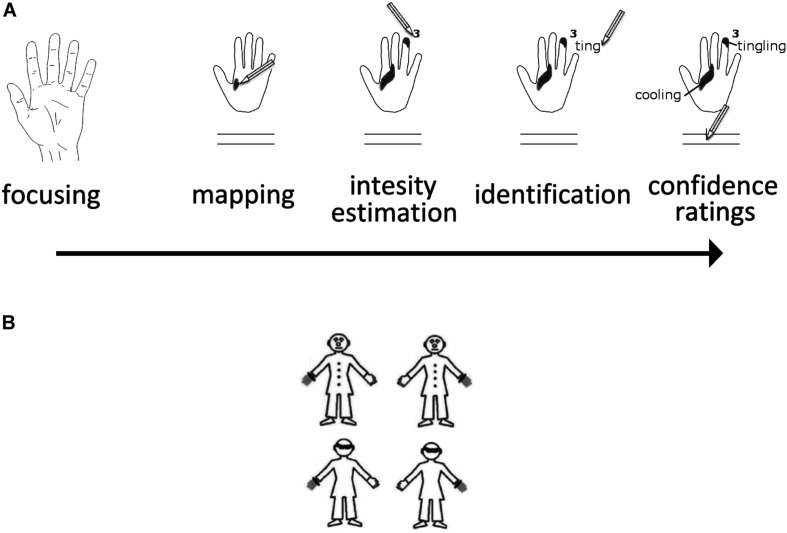
**(A)** Illustration of the SPS Paradigm. **(B)** The four stimuli used in the OBT task are shown here.

Participants completed the task at a desk in a quiet, normally lit room with an ambient temperature of 20–23°C. Once they were ready, SPS were introduced as normal phenomena, and to give the participants some idea of what they could expect SPS to feel like, they were given a list of 11 sensations most likely to be felt (beat/pulse, itch, tickle, numbness, skin stretch, tingling, warming, cooling, muscular stiffness, flutter, and vibration). The list used in the SPS protocol was based on the one used originally by Ochoa and Torebjörk (1983), Macefield et al. (1990), Naveteur et al. (2005). Participants were asked to remove any jewelry from their hands and wrists, and to roll up their sleeves to ensure the wrist was not covered. They then had to wash their hands with an antiseptic gel for 15 seconds (Aniosgel^®^ 85 NPC, ≈3 ml per participant), in order to remove any external surface agents that could interfere with the task, and to ensure a homogenous glabrous skin surface across all participants. The test began 15 seconds after the participants finished washing their hands. A protocol was given to each participant. It contained the standardized reduced maps of each hand shown palm up (with a distance of 11.2 cm between the tip of the middle finger and the palm/wrist border). Below each map, the list of 11 SPS was provided, along with two visual analogue scales (i.e., two 10-cm continuous horizontal lines without markers at each ends) for the purposes of confidence rating. A pencil and a 25 × 25 cm piece of smooth white fabric were also given to each participant. Once participants had familiarized themselves with the materials, the task began. The protocol and the pencil were placed away from the participants on the desk to prevent any interference from visual stimuli. Each hand was tested once in a balanced order across the participants. The participants had to sit with their back supported by the backrest of their chair. The leg ipsilateral to the tested hand was turned outwards by about 60 degrees from the midline. Participants placed the white cotton fabric on their thigh, with the tested hand resting on it, palm up, with fingers spaced slightly apart. Only the dorsal part of the hand was in contact with the fabric. The hand not being tested was allowed to hang down on the outer side of the chair. A “start” signal was given verbally by the experimenter, marking the beginning of each test. Participants were asked to gaze at the tested hand for 10 seconds, focusing their attention on the whole hand so that they could detect any sensations that might occur. They were informed beforehand that there was a possibility they would not perceive any sensations, and this is also normal. A “stop” signal given by the experimenter marked the end of the focusing period. Participants were immediately asked to use the protocol to report whether they had detected any sensations on the tested hand, and if they had, to (a) map the extent and location of the sensations by shading on the map of the tested hands the areas where they perceived sensations, (b) estimate the perceived intensity of each sensation according to a 10-point scale (1 = just perceptible; 10 = very intense but not painful), (c) identify the sensations using the list of descriptors. They were allowed to choose more than one descriptor and to add descriptors to the list based on what they detected, and (d) indicate their degree of confidence in the location and extent of the perceived sensations on the two visual analogue scales (ranging from “not confident” to “very confident”). The task lasted approximately 10 minutes, and depicted in Figure 1A.

### OBT Task

The stimuli and the procedure were taken from a previous study on own-body transformations (Arzy et al., 2007). Participants were presented with a human figurine subtending a visual space of 5.0° × 6.1° angles. The figurine could be front-facing or back-facing, and had either the left or the right hand marked, such that it appeared to be wearing a gray glove with a black ring at the wrist (Figure 1B). Stimuli appeared for 200 ms in the center of the computer screen with an inter-stimulus interval of 2000 ms. Participants were asked to make right/left judgments while imagining themselves in the body position of the figure, taking its spatial perspective. This requires mental own-body positioning that can be achieved through mental transformations (O.Blanke, 2005). Responses were given by pressing two predefined buttons on the computer keyboard using the index finger of each hand. Participants were asked to respond as quickly and accurately as possible but always to perform the requested mental transformation before giving the response. A total of 120 trials were presented (60 per facing condition), and the session was preceded by a 10-trial training session. The facing (front vs. back) condition and the gray hand location (left vs. right) were presented randomly and equiprobably. Response times and correct responses were recorded by the computer.

## Results

### SPQ

The mean scores for each of the three factors of the SPQ were 7.1 (SD = 5.7) for cognitive-perceptual, 7.9 (SD = 5.7) for social-interpersonal, and 5.1 (SD = 3.6) for disorganized. The mean score for the full SPQ scale was 20.1 (SD = 12.1). Internal consistency of each subscale for the present sample was good: cognitive-perceptual α = 0.87, social-interpersonal α = 0.82, disorganized α = 0.91. The internal consistency of the total score was excellent, with α = 0.93.

### OBT Task

Response times (RT) below 150 ms and over 2000 ms were dismissed as reflecting anticipation and inattention, respectively. Discarded trials accounted for less than 2% of the total number of trials. Response times for correct trials and the number of correct trials were submitted to *t*-tests with the facing condition (front vs. back) as the within factor. As in previous investigations, RTs were faster in the back-facing condition (mean RT = 790 ms, SD = 205 ms) than in the front-facing condition (mean RT = 917 ms, SD = 265 ms; *t*(54) = 7.23, *p* < 0.001, Cohen’s *d* = 0.96). And as in previous investigations, no differences were found between the two facing conditions as far as the number of correct responses was concerned (front-facing mean = 56.6, SD = 7.7; back-facing mean = 57.4, SD = 2.7; *t*(54) = 0.75, *p* > 0.45, Cohen’s *d* = 0.1). Since both facing conditions evaluate own-body mental transformations in a similar way, the mean RT and the mean number of correct responses were used for subsequent analyses, as in previous papers (O.Blanke, 2005; Mohr et al., 2006).

### Topography of SPS Frequency

**FIGURE 2:**
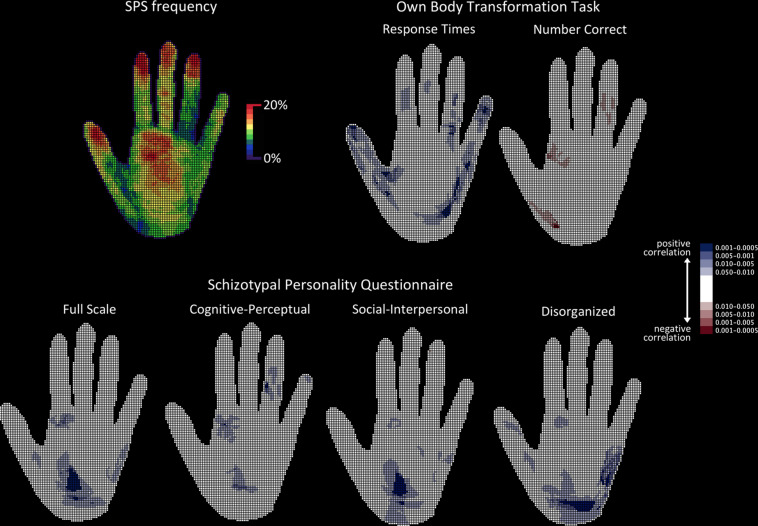
Topographical analyses of SPS frequency. The hand is shown palm up. **Top left:** Raw frequency of SPS showing the proximo-distal gradient. **Top right:** Probability maps of the topographical cell-wise point-biserial correlation analysis carried out between response times and number of correct responses obtained in the OBT task and the presence of SPS. **Bottom:** Probability maps of the topographical cell-wise point-biserial correlation analysis carried out between individual scores in the SPQ (total scale and each of the three individual SPQ factors). For all probability maps, only significant clusters are presented. Bluish cells denote significant positive correlations, while reddish cells denote significant negative correlations. Color shading on the probability map represents different probabilities.

Maps filled by participants with shaded areas were projected onto a 140 × 140 mm grid, with a 1 mm × 1 mm resolution, and converted into binary code (0 = nil, 1 = shaded cell). This generated two binary maps per participant (i.e., right and left hand), with a total number of 110 maps. By superimposing these maps, a frequency map was obtained, in which each cell value represented the percentage of participants who had shaded it. The presence of a distal-to-proximal gradient in frequency was observed (Figure 2, top left), with the surface of the reported SPS relative to the surface of each anatomical segment of the hand being, respectively, 14.3% for the distal phalanx, 10.0% for the intermediate phalanx, 8.8% for the proximal phalanx, and 10.4% for the palm. This suggests that the SPS task was successful.

### SPS Frequency and Spatial Correlations

This analysis was carried out to detect any significant effects in the spatial distribution of SPS as a function of the SPQ scores and OBT task performance. In order to achieve a cell-by-cell analysis, SPQ score, RT, and number of correct responses obtained in the OBT task were used to fill two maps (right and left hand) for each participant. A point-biserial cell-wise correlation between the presence of SPS and the SPQ and OBT measures was performed, with *N* = 110 maps, degrees of freedom = 108, and the probability threshold set at 0.05, one-tailed due to the oriented hypotheses. The point-biserial correlation is a special case of the Pearson product-moment correlation that follows the *t*-distribution and in which one variable is dichotomous (here, 0 = non-shaded cell; 1 = shaded cell) and the other variable is continuous. Afterwards, the correlation maps obtained were converted into binary maps (0 = non-significant cell, 1 = significant cell), for the application of a spatial scan procedure for binary data (Kulldorff, 1997). This consists of a circular window that scans, detects, and localizes significant clusters in a step-wise manner. Based on previous studies, a radius of 6 cells was chosen, containing 113 cells and corresponding to a surface area of 1 cm^2^ on a real hand (Michael et al., 2012). After 999 runs of the Bernoulli (binomial) model, all detected and localized clusters were significant to at least the *p* < 0.001 level bicaudal. Finally, the 95% two-sided asymmetrical confidence interval was also computed (Altman et al., 2000) for the significance of the overall percentage area of the hand covered by previously detected reliable clusters.

### SPS Frequency and OBT Task

Only positive correlations were found between SPS frequency and mean RT obtained in the OBT task. The *r*-values ranged from 0.20 to 0.30, and the corresponding *p*-values from 0.02 to 0.0007 one-tailed. Significant clusters detected by the spatial scan statistic were distributed over the fingers and the palm (both thenar and hypothenar eminences) and covered 22.8% (95% confidence interval: 21.6% to 24.1%) of the total surface area of the hand. Conversely, only negative correlations were found between SPS frequency and the mean number of correct responses obtained in the OBT task (*r*-values range = −0.19 to −0.28; *p*-values range = 0.025 to 0.001 one-tailed). Significant clusters detected by the spatial scan statistic were located in the fingers and the palm and covered 4.2% (95% confidence interval: 3.7% to 4.9%) of the total surface area of the hand. The correlations between SPS frequency and the OBT task are presented in Figure 2.

### SPS Frequency and SPQ Scores

Only positive correlations were observed between SPS frequency and total SPQ score (*r*-values range = 0.21 to 0.28; *p*-values range = 0.015 to 0.0015 one-tailed), as well as between SPS frequency and each of the SPQ subscales (cognitive-perceptual *r*-values range = 0.20 to 0.26; *p*-values range = 0.02 to 0.0025 one-tailed; social-interpersonal *r*-values range = 0.16 to 0.285; *p*-values range = 0.05 to 0.001 one-tailed; disorganized *r*-values range = 0.16 to 0.30; *p*-values range = 0.05 to 0.0007 one-tailed). Significant clusters of correlations with total SPQ score subtended an overall area of as much as 13.5% of the hand (95% confidence interval: 12.5% to 14.6%) and were all located in the palm. Significant clusters of correlations with the cognitive-perceptual SPQ subscale score subtended an area of 7.3% of the hand (95% confidence interval: 6.6% to 8.1%) and were located in the fingers and the palm. Significant clusters of correlations with the social-interpersonal SPQ subscale score subtended an area of 11.1% of the hand (95% confidence interval: 10.1% to 12.1%) and were located in the palm. Finally, significant clusters of correlations with the disorganized SPQ subscale score subtended an area of 18.2% of the hand (95% confidence interval: 17.1% to 19.4%) and were located in the palm and the thumb.

### Other Parameters

Other SPS parameters, i.e., mean perceived intensity, confidence in location and extent, total number of disjoined areas, spatial extent per area, and variety of SPS, were submitted to multiple linear regression analyses with OBT task performance (mean RT and number of correct responses) and SPQ scores (cognitive-perceptual, social-interpersonal, disorganized) as predictors. Age, gender, and BMI were also used as predictors, since previous research has shown that they may influence the perception of SPS (Michael and Naveteur, 2011; Michael et al., 2015; Naveteur et al., 2015). No significant effects were found (Table 1). A second, independent analysis was conducted in order to assess whether schizotypy interacted with performance in the OBT task. This moderation relationship was assessed through a regression analysis where the total SPQ score ^∗^ RT in the OBT task, and the total SPQ score ^∗^ number of correct responses in the OBT task were used as predictors, and each of the abovementioned SPS parameters was used as dependent variable. The total SPQ score ^∗^ number of correct responses in the OBT task significantly and negatively predicted confidence in location (β = −38.05, SEM = 17.68, *t* = 2.15, *p* < 0.037) meaning that the higher the degree of schizotypy and the better the performance in the OBT task, the lower the confidence in the location of SPS.

**TABLE 1:** Results of the regression analyses (β and SEM) carried out with the parameters and categories of spontaneous sensations as dependent variables.

	**SPS parameters**						**SPS categories**				
	**Intensity**	**Confidence**	**Confidence**	**Number of**	**Spatial extent**	**Variety**	**Thermal**	**Deep**	**Surface**	**Paresis-Like**	**Pain-Like**
		**in location**	**in extent**	**disjoined areas**	**per area**						
**Demographics**											
Age	−0,005 (0,15)	−0,103 (0,15)	−0,052 (0,15)	0,105 (0,15)	−0,097 (0,16)	0,041 (0,15)	−0,087 (0,14)	0,147 (0,16)	0,037 (0,16)	0,112 (0,15)	0,181 (0,15)
Gender	−0,198 (0,18)	−0,101 (0,18)	0,033 (0,17)	−0,059 (0,18)	0,161 (0,19)	−0,205 (0,17)	0,078 (0,17)	−0,098 (0,18)	0,02 (0,18)	0,055 (0,18)	0,004 (0,2)
**BMI**	−0,179 (0,15)	−0,216 (0,15)	−0,296 (0,15)	−0,13 (0,15)	0,048 (0,15)	−0,241 (0,14)	−0,14 (0,14)	−0,021 (0,16)	−0,096 (0,15)	−0,157 (0,15)	−0,142 (0,14)
**SPQ**											
Cognitive-perceptual	0,052 (0,18)	0,085 (0,18)	0,111 (0,17)	0,145 (0,18)	−0,089 (0,19)	0,044 (0,17)	0,372 (0,17) *	0,126 (0,18)	−0,043 (0,18)	−0,162 (0,17)	−0,09 (0,17)
Social-interpersonal	0,045 (0,18)	−0,074 (0,18)	0,066 (0,18)	−0,005 (0,19)	0,073 (0,19)	0,02 (0,17)	0,225 (0,17)	−0,01 (0,18)	0,058 (0,19)	0,391 (0,18) *	−0,123 (0,18)
Disorganization	0,103 (0,18)	0,179 (0,18)	0,081 (0,18)	0,145 (0,19)	0,033 (0,19)	0,131 (0,17)	−0,293 (0,17)	−0,101 (0,19)	0,205 (0,19)	−0,176 (0,18)	0,284 (0,18)
**OBT task**											
Response Times	−0,156 (0,16)	−0,018 (0,16)	0,082 (0,15)	0,004 (0,17)	0,109 (0,16)	−0,019 (0,15)	−0,151 (0,15)	−0,037 (0,16)	0,038 (0,16)	0,022 (0,15)	0,322 (0,15) *
Number correct	0,038 (0,15)	−0,005 (0,15)	−0,071 (0,15)	0,08 (0,16)	0,008 (0,16)	−0,159 (0,15)	−0,095 (0,14)	−0,06 (0,16)	0,071 (0,16)	−0,158 (0,15)	0,041 (0,15)

SPS, Spontaneous Sensations; BMI, Body MassIndex; SPQ, Schizotypal Personality Questionnaire; OBT task, Own Body Transformation task. *Significant prediction.

### Types of Sensations

All SPS types were reported at least once and some participants even reported other types of sensations, such as pins and needles, weightiness, and compression. The reported SPS types were sorted into five categories based on previous studies (Beaudoin and Michael, 2014): thermal (warming and cooling), deep (beat/pulse and muscle tension), paresis-like (numbness and weightiness), surface (tickle, stretch, tingling, flutter, vibration, and compression) and pain-like (pins and needles, and itch). SPS types were not distributed uniformly across these five categories (χ*^2^*(4) = 218.4, *p* < 0.001). Surface-type SPS were most likely to be perceived and reported (55.2%), followed by deep (20.8%), thermal (13.2%), paresis-like (9.6%), and pain-like sensations (1.2%). Each of the five categories of sensations, as well as the total number of reported SPS, were submitted to multiple linear regression analyses with OBT task performance, SPQ scores, age, gender, and BMI as predictors (Table 1). The SPQ cognitive-perceptual scale positively predicted the number of thermal sensations (β = 0.37, SEM = 0.17, *t* = 2.18, *p* < 0.035), and the social-interpersonal scale positively predicted the number of paresis-like sensations (β = 0.39, SEM = 0.18, *t* = 2.13, *p* < 0.039). Finally, mean RT from the OBT task positively predicted pain-like sensations (β = 0.32, SEM = 0.16, *t* = 2.04, *p* < 0.049). The same moderation analysis as before was conducted on the types pf sensations. No significant effect was found.

### Correlation Between the SPQ and the OBT Task

Pearson correlations were carried out between the SPQ scores and OBT task performance, with *N* = 55 and degrees of freedom = 53. No significant correlations were found (all *r* < 0.18, all *p* > 0.20).

## Discussion

To our knowledge, along with interoception, the perception of SPS is a domain that seems related to that aspect of embodiment that is the direct and immediate experience of our own body in the first-person perspective. The difference between interoception and SPS perception is that the former targets the monitoring of internal bodily signals (Vaitl, 1996) while the latter expands to *feeling* one’s own body and its boundaries (Naveteur et al., 2015; Michael et al., 2017). However, both are encompassed by the broader definition of interoception [(Bud) Craig, 2003] and are seemingly related phenomena (Michael et al., 2015). The aim of this study was to investigate the relationship between the perception of SPS and embodiment, since the literature provides mostly indirect evidence of this (Michael and Naveteur, 2011; Bauer et al., 2014b; Michael et al., 2017). This was achieved by combining an SPS perception task, an experimental own-body mental transformation task (OBT task), and a questionnaire assessing schizotypal personality traits.

### Embodiment, Disembodiment, and SPS

Since embodiment is related to the experience of one’s own body in the first-person perspective, changing perspective by mentally rotating the body image in order to judge whether it matches a visually presented figurine is challenging. Indeed, complex body-related mechanisms are required (Blanke, 2005; Kaltner et al., 2014; van Elk and Blanke, 2014), such as flexible control of self-perspective-taking and disembodiment (Arzy et al., 2007), and alignment of somatosensory and vestibular information (Zacks et al., 1999; van Elk and Blanke, 2014; Gardner et al., 2017). Therefore, better performance in the OBT task (i.e., shorter RT and higher accuracy) suggests that changing self-perspective and disembodying is achieved more easily. The ease with which this task is completed thus relates to loose embodiment: a proneness to experiencing out-of-body experiences (Blanke, 2005), in which the sense of spatial unity between self and body is abnormal since the self is not experienced as residing within the limits of one’s body (Blanke et al., 2004; Blanke and Arzy, 2005). The results paint a quite coherent picture. The frequency with which SPS were perceived correlated positively with RT in the OBT task. Since performing the OBT task quickly requires flexible control of disembodying and self-perspective-taking, and since a higher RT in the OBT task suggests greater difficulty in completing the task, this correlation suggests that difficulties in disembodying voluntarily and mentally transforming one’s own body facilitates *feeling* oneself. Similarly, the frequency with which SPS were perceived correlated negatively with accuracy in the OBT task. Since performing the OBT task correctly requires flexible control of disembodying and self-perspective-taking, and since greater accuracy in the OBT task suggests less difficulty in completing the task, this correlation suggests that the ability to easily disembody and mentally transform one’s own body decreases the sense of *feeling* oneself. Taken together, these results suggest that the perception of untriggered bodily sensations is related to embodiment and that embodiment is required in order to correctly *feel* oneself. The ability to disembody easily reduces this *feeling*. Since the correlation between OBT task performance and SPS perception was not perfect, the present experiment does not allow us to draw conclusions as to which of the processes involved in the OBT task determine the perception of SPS, or even to what degree. For instance, processes of spatial mental manipulation, attention, and simulation of sensory and vestibular information have been advanced as determinants of the ability to align one’s own body orientation to that of a third party and to perspective-taking (Gardner et al., 2017, 2018; Pesimena et al., 2019).

### Schizotypy and SPS

Our investigation into the way in which SPS contribute to embodiment also involved a self-completed schizotypal personality questionnaire, the SPQ (Raine, 1991). Schizotypal personality is considered to be a model for the study of schizophrenia spectrum disorders (Sass and Parnas, 2003; Nelson et al., 2012; Barrantes-Vidal et al., 2015), in which disruptions to the basic sense of embodiment are viewed as key symptoms (Stanghellini et al., 2012; Ferri et al., 2014). Therefore, assessing the relationship between schizotypal personality and the perception of SPS can provide insights not only into sense of self and embodiment, but into their disturbances too. Schizotypal personality is associated with a proneness to experiencing self-related disturbances (Mohr et al., 2006; Arzy et al., 2007), altered self-other boundaries (Di Cosmo et al., 2018; Ferroni et al., 2020), difficulties in controlling perspective-taking (Langdon and Coltheart, 2001), and unusual, exaggerated and vivid perceptual experiences involving the body (McCreery and Claridge, 2002; Sass and Parnas, 2003; Ettinger et al., 2015; Van Doorn et al., 2018). It was thus expected that higher schizotypal traits would correlate with the perception of SPS, signaling altered body boundaries (Di Cosmo et al., 2018) and difficulties in controlling and changing self-perspective (Mohr et al., 2006). The results showed that proneness to the cognitive-perceptual, social-interpersonal, and disorganized factors of schizotypy is sufficient to produce enhanced perception of SPS, as expected. The literature mostly points to the cognitive-perceptual factor as being associated with body-related unusual phenomena (Raine, 1991; Raine et al., 1994; Mohr et al., 2006; Arzy et al., 2007; Barrantes-Vidal et al., 2015; Ettinger et al., 2015). However, our findings are corroborated by some studies, which have shown that other factors may also relate to body-related experiences (Van Doorn et al., 2018), suggesting that altered sense of self is probably a hallmark of schizotypal personality as a whole. This is backed up by the interacting effect found between the total SPQ score and performance in the OBT task which suggests that despite more vivid perception of SPS due to both higher risk for psychosis and to the ease with which one disembodies and mentally transforms own body (as shown separately through the frequency of SPS), localizing SPS is less certain. This strengthens the idea of blurred bodily boundaries in schizotypy and also tallies well with the idea that abnormal bodily experiences may be a presenting symptom of schizophrenia spectrum disorders (Sass and Parnas, 2003; Quinones, 2009; Stanghellini et al., 2012). At this point it is also possible to hypothesize that, as compared to a control sample, patients with schizophrenia would report larger sensitive zones of SPS as a reminiscence of the relationship found between SPS perception and the degree of schizotypy. Also, lower degrees of confidence in their location and extent are to be expected as a result of altered perceptual experiences and malleable body frontiers (Sass and Parnas, 2003; Di Cosmo et al., 2018; Ferroni et al., 2019).

The overall similar topography of correlations between SPS frequency and each of the factors subtending schizotypal personality, as well as the fact that the total SPQ score seems to combine these patterns, backs the idea that SPS perception captures subtle aspects of body-related unusual experiences, as has already been shown with different kinds of syndromes (Borg et al., 2015; Echalier et al., 2020), making it possible to relate schizotypal personality as a whole to alterations in *feeling* oneself. The exact origins of aberrant bodily perception in schizotypal personality are not known, but there is evidence suggesting that somatosensory cortical processing is modified (Lenzenweger, 2000; Chang and Lenzenweger, 2005), that the remapping of environmental stimuli in the body space is altered (Ferri et al., 2016), and that multisensory integration necessary to the perception of the self and distinction from others may be involved (Burrack and Brugger, 2005; Asai, 2016; Di Cosmo et al., 2018; Ferroni et al., 2020). All these aspects are essential for embodiment (Gallace and Spence, 2010; Sakson-Obada et al., 2018; Manos Tsakiris, 2010, 2017), since they allow us to establish the nature and location of perceived sensations, as well as the boundaries between the self and the external world (Bretas et al., 2020). We believe this is where embodiment meets the perception of SPS, one function of which is to provide information on the spatial boundaries of the body. Indeed, perceiving the boundaries of the body is central to distinguishing it from the world and others, an aspect that is altered in schizotypy and, generally, on the schizophrenia spectrum (van der Weiden et al., 2015; Asai, 2016).

### Origins of Feeling Oneself

From a phenomenological perspective, there is no doubt that perceiving SPS is *feeling* oneself as a bodily entity that is distinct from others and from nearby objects, and characterized by somatic sensations of various qualities experienced from a first-person perspective. But direct evidence of this is still required. The relationship between SPS and the OBT task backs the idea that changing perspective is detrimental to the perception of SPS and that, consequently, SPS contribute to experiencing sensations from the first-person perspective.

According to theories of self-awareness, there is an ever-present background of bodily sensations that may be the basis for constructing the continuity of the experiencing self (Kinsbourne, 1998). This background would maintain the integrity of the body and would be partly responsible for how the self is perceived (Sakson-Obada et al., 2018). Orienting and focusing attention toward the self would enable the representation of one’s own body or body parts to arise (Michael and Naveteur, 2011; Michael et al., 2012) and be maintained in consciousness (Wolpert et al., 1998), and this may be the representation over which attention is shifted (Kinsbourne, 1998). The postulated theory behind these accounts is that the sense of self arises due to interaction between the systems and brain structures that contain information about the body (e.g., somatosensory and interoceptive) and those that control attention. Interestingly, both attention and body-related processes are altered in schizotypy and schizophrenia spectrum disorders (Lenzenweger, 2000; Chang and Lenzenweger, 2005; Moe and Docherty, 2014; Ettinger et al., 2015; Ferri et al., 2016; Michael et al., 2020), one consequence of which may be aberrant bodily perceptions. It is not absurd to imagine that disordered attention processes operating over body representations are the origin of aberrant perceptual phenomena, such as body-related illusions, tactile and bodily hallucinations, and bodily perceptual distortions (Behrendt, 2006). We believe that when attention operates normally over the somatosensory and interoceptive cortices, SPS may be perceived (Michael et al., 2012, 2015), particularly given that activity in the primary and secondary somatosensory cortices does relate to these phenomena (Bauer et al., 2014a,b). However, in cases of altered attention, this may lead to distorted perception of such sensations (Borg et al., 2015), body perceptual alterations (Echalier et al., 2020), phantom limbs (Kinsbourne, 1998), asomatognosia (Berlucchi and Aglioti, 1997), and tactile hallucinations (Behrendt, 2006). Another theory would be that the spontaneous cortical activity that confers the characteristics of normal SPS may be easily mistaken as coming from outside the body if the internal origin is not correctly appreciated (Larøi and Woodward, 2007). In this last case, not only may bodily hallucinations arise, but the implicit bodily experience one has of one’s own body may be altered, giving rise to experiences such as abnormal feelings of transformation (Stanghellini et al., 2014).

### Limitations

One limitation of the present study is the unbalanced number of male and female participants, with female participants outnumbering males. This mostly stems from the fact that participants were undergraduate students in Social Sciences and Humanities where, at least in France, female students are as much as four times more numerous than male students. Furthermore, within the 15 years of scientific investigation of SPS, we found that the probability of males being excluded from the study is high because of regular use of substance and drugs. An unbalanced sample may prevent from detecting gender-related effects, and indeed previous investigations found such gender differences in both the OBT task (Mohr et al., 2006) and SPS (Naveteur et al., 2015). Yet, except from the fact that gender differences were not one of the main factors under investigation in the present study, research showed that the SPS patterns observed in female participants (Naveteur et al., 2015) are more representative of overall performance (i.e., mixing up male and female participants) and can be more easily generalized.

An observation that may appear surprising at first glance is the complete absence of correlation between the OBT task and the SPQ. Previous research found that the higher the schizotypal traits, the slower the performance in this task (Mohr et al., 2006). However, the authors did not use a general schizotypal personality tool, as was used here. They instead used a questionnaire focusing on perceptual aberrations that included multiple assessments of body-related phenomena (Chapman et al., 1978). A closer inspection of the SPQ reveals that unusual body-related experiences are assessed in just two questions (one related to the feeling of presence and one to deformations of faces seen in a mirror). The SPQ therefore covers more global aspects of schizotypy, and this may be why no relationship was found with the OBT task. Interestingly, as explained above, both the OBT task and the SPQ correlated with SPS, despite not correlating with each other, suggesting that SPS may capture those specific aspects of each measure that relate to sense of self and the way in which the body and its boundaries are felt. The interaction found between the total SPQ scale and performance in the OBT task in predicting confidence in the location of SPS may reflect this idea.

Another limitation is that only the spatial distribution and some types of SPS were found to relate with OBT task performance and SPQ score. The remaining SPS parameters did not. This is not surprising, however, since previous research has shown that these parameters may be less sensitive, probably due to significant between-subject variability. One possible reason is the moderate size of our sample. Indeed, as power analyses suggest, the present sample is enough to detect the most common characteristic of SPS, which is the proximo-distal gradient, but it may not suffice for revealing more subtle effects that also may greatly vary between participants.

## Conclusion

The present study provides a coherent picture of the relationship between *feeling* oneself, as assessed through the perception of SPS, and embodiment, as assessed through the OBT task and as seen in the schizotypal personality. Embodiment is needed in order to correctly feel and sense oneself. These findings complement previous research, which found that SPS may be interoceptive in nature (Michael et al., 2015), and show that feeling oneself is not only related to the perception of internal visceral signals, but also to the perception of tactile sensations that arise on the surface of the body without any external triggers. We believe that this study helps us understand normal and disordered bodily awareness and that the methodology associated with the assessment of SPS could be used as a research tool for addressing issues that are difficult to induce experimentally, such as tactile and bodily hallucinations, and phantom limbs. Finally, dissemination of the findings could help therapists to better apprehend disturbances of embodiment and their relationship to the schizophrenia spectrum, and develop behavioral methods and practices for the treatment and prevention of disordered body image and the self. For instance, giving precise information about the nature of sensations that are spontaneously perceived at rest may constitute a prevention practice, while helping patients with disorders of the self to focus on relevant bodily and interoceptive sensations, and providing them with techniques to scan their body and interpret those sensations may contribute to reduce feelings of anxiety.

## Data Availability Statement

The datasets presented in this study can be found in online repositories. The names of the repository/repositories and accession number(s) can be found in the article/supplementary material.

## Ethics Statement

This study involving human participants was reviewed and approved by the Comité de Protection des Personnes Sud-Est II (reference number IRB Groupement Hospitalier Est, 59 boulevard Pinel, 69500 Bron, France. The patients/participants provided their written informed consent to participate in this study.

## Author Contributions

GM supervised the research, conducted the statistical analysis, and wrote the majority of the manuscript. DG and ET created the code for the OBT task and collected and analyzed data. SS and MC provided technical assistance. All the authors made a significant contribution to drafting the article and revising it critically for important intellectual content, discussed the results, commented on the manuscript, and gave their final approval of the version for publication, contributed to the article and approved the submitted version.

## Conflict of Interest

The authors declare that the research was conducted in the absence of any commercial or financial relationships that could be construed as a potential conflict of interest.
